# Electroacupuncture reshapes the microbial co‐occurrence networks related to the behavioral and psychological symptoms of dementia in Alzheimer's disease

**DOI:** 10.1002/imo2.70035

**Published:** 2025-07-02

**Authors:** Fu‐You Su, Chia‐Min Lin, Chao Liu, Yiqin Yao, Hui Wang, Chunxue Zhang, Wei Yi, Nenggui Xu

**Affiliations:** ^1^ South China Research Center for Acupuncture and Moxibustion Medical College of Acupuncture‐Moxibustion and Rehabilitation, Guangzhou University of Chinese Medicine Guangzhou China; ^2^ Department of Electrical and Computer Engineering Tamkang University Taiwan China; ^3^ Nanjing First Hospital, Nanjing Medical University Nanjing China; ^4^ The First Affiliated Hospital of Guangzhou University of Chinese Medicine Guangzhou China

## Abstract

Microbial keystone species and gut microbiota composition are highly variable during the pathological development of the behavioral and psychological symptoms of dementia (BPSD) in Alzheimer's disease (AD). Age stratification reveals stage‐specific gut microbial signatures in AD‐related BPSD. This study highlights the efficacy of electroacupuncture in regard to altering the intestinal microbial landscape in AD‐related BPSD and provides novel insights into the application of phased targeted electroacupuncture interventions in the future.

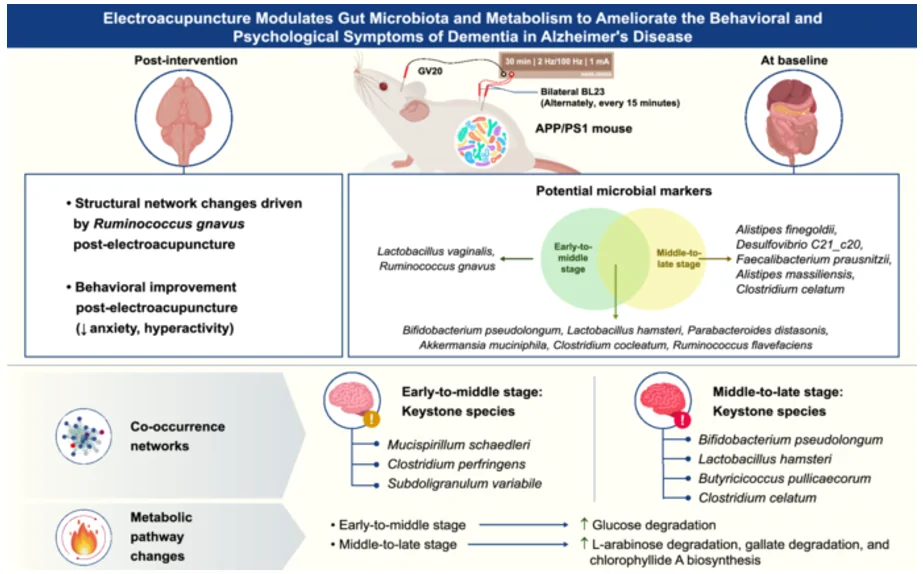

Although the gut microbiota plays a crucial role in the pathogenesis of Alzheimer's disease (AD), its association with the behavioral and psychological symptoms of dementia (BPSD) in AD has yet to be investigated. BPSD belongs to the spectrum of neuropsychiatric syndromes (NPS), encompassing more than 12 complex symptoms divided into four major categories [1]. The stratification of BPSD has evolved rapidly over recent years. AD‐related BPSD is a multifaceted process that occurs along the AD continuum. Unlike the mild behavioral impairment observed in the prodromal and early stages of AD and the psychiatric disorders that occur in the late stage of AD, BPSD represents a late‐onset NPS that is most commonly observed in the early‐to‐middle and middle‐to‐late stages of AD. Only a paucity of information is currently available relating to the mechanisms underlying the regulation of microbial composition, co‐occurrence network topologies, and predictive metabolic pathways in AD‐related BPSD. In this study, we investigated the effects of electroacupuncture on the dynamics of core microbiota and keystone species in the microbial co‐occurrence networks of AD‐related BPSD, as well as their crosstalk with the predictive metabolic pathways. To the best of our knowledge, our research represents a pioneering approach in this area and provides a valuable foundation for further investigation.

## 1 RESULTS AND DISCUSSION

### Profiles of disease‐discriminatory bacterial taxa and optimal microbial markers

**Figure 1:**
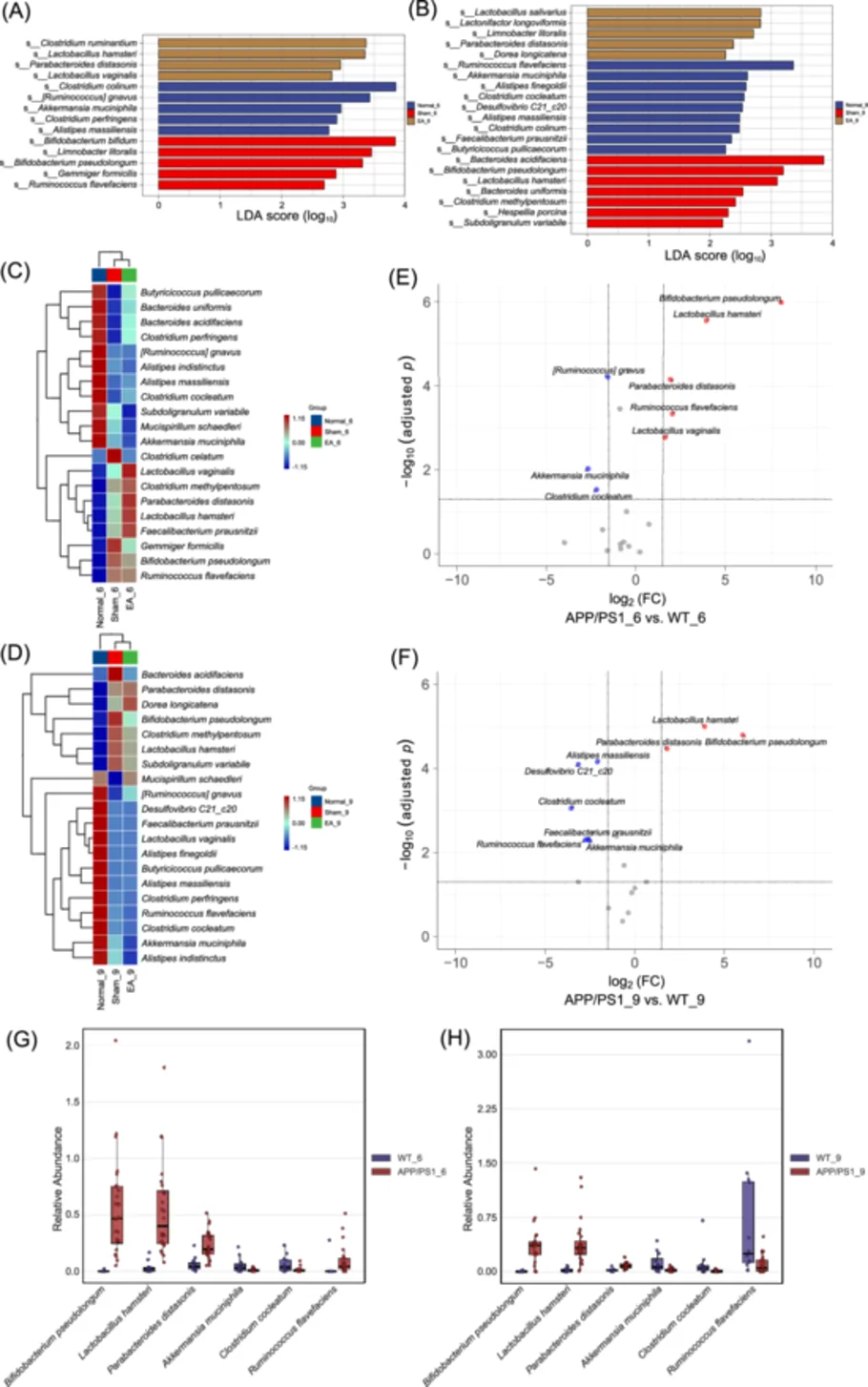
Identification of microbial markers that discriminate APP/PS1 mice from WT mice. Linear discriminant analysis (LDA) plots showing differentially abundant taxonomic units between the 6‐month‐old (A) and 9‐month‐old (B) groups. The *x*‐axis represents the LDA score on the Log_10_ scale, with a threshold of 2 for both ages. The *y*‐axis represents the significantly differential taxa, and the letter S in front of each name stands for the “species.” Heatmaps showing hierarchical clustering relationships of gut microbiota in the 6‐month‐old (C) and 9‐month‐old (D) groups. Volcano plots highlighting differences in the abundance of gut microbiota between 6‐month‐old (E) and 9‐month‐old (F) APP/PS1 and age‐matched wild‐type (WT) mice. The red and blue dots represent species that are up‐ and downregulated, respectively, in APP/PS1 mice compared with WT mice. Boxplots showing variations in the disease‐discriminatory species shared by 6‐month‐old (G) and 9‐month‐old (H) APP/PS1 mice.

Our analyses demonstrated that *Bifidobacterium pseudolongum, Lactobacillus hamsteri*, and *Parabacteroides distasonis* were significantly enriched (*p*_adj_ < 0.05), while *Akkermansia muciniphila* and *Clostridium cocleatum* were significantly depleted (*p*_adj_ < 0.05) in both 6‐ and 9‐month‐old APPswe/PS1δE9 (APP/PS1) mice (Figures 1A–H, S1A,B, S2A,B, and Tables S1–S4). Due to the marked variation in the abundance of *Ruminococcus flavefaciens* in both age groups of wild‐type (WT) mice, the comparison between AD and WT mice exhibited an opposing trend: a higher abundance in 6‐month‐old APP/PS1 mice but a lower abundance in 9‐month‐old APP/PS1 mice when compared to age‐matched WT mice (Figure1G,H). Furthermore, the specific microbial markers that distinguished 6‐month‐old APP/PS1 mice from age‐matched WT mice included *Lactobacillus vaginalis* and *Ruminococcus gnavus*, while those that distinguished 9‐month‐old APP/PS1 mice from age‐matched WT mice included *Alistipes finegoldii*, *Desulfovibrio C21_c20*, *Faecalibacterium prausnitzii*, *Alistipes massiliensis*, and *Clostridium celatum* (Tables S3 and S4).

Next, we used a Random Forest algorithm, a powerful machine learning paradigm with an ensemble of decision tree models, to evaluate the importance scores of potential microbial markers for AD‐related BPSD. Analysis revealed that the optimal microbial markers for BPSD in the early‐to‐middle stage of AD were *B. pseudolongum*, *L. hamsteri*, *P. distasonis*, *L. vaginalis*, *A. muciniphila*, *R. flavefaciens*, and *Clostridium perfringens* (Figure S2C), while those for BPSD in middle‐to‐late stage AD were *A. muciniphila*, *P. distasonis*, *Clostridium Methylpentosum*, *L. hamsteri*, *B. pseudolongum*, and *A. finegoldii* (Figure S2D).

### Electroacupuncture reshaped microbial composition and ameliorated behavioral outcomes during the open field test (OFT)

Heatmap, Linear discriminant analysis Effect Size, Volcano, and Orthogonal partial least squares‐discriminant analysis revealed distinct clustering relationships, microbial composition, and topological distribution across all groups post‐intervention (Figures S3 and S4). The OFT demonstrated that the total distance traveled and the frequency of central zone crossings by 6‐ and 9‐month‐old APP/PS1 mice were significantly greater than those of age‐matched WT mice (*p*_adj_ < 0.05; Figure S5A–D). Furthermore, the distance traversed in the central zone by 6‐ and 9‐month‐old APP/PS1 mice was greater than that by age‐matched WT mice, but the difference was not significant (Figure S5E,F). These data indicate that APP/PS1 mice exhibit characteristics commonly observed in AD‐related BPSD, including positive symptoms such as agitation, anxiety, hyperactivity, and aimless wandering. The duration of stay in the central zone did not significantly differ between APP/PS1 and WT mice in either age group (Figure S5G,H). Notably, all the aforementioned indices decreased significantly (*p*_adj_ < 0.05) in the 6‐ and 9‐month‐old electroacupuncture (EA) groups post‐intervention compared to the values observed in age‐matched Sham groups (Figure S6A–H). These indices, except for the total distance traveled (Figure S6A,B), decreased significantly (*p*_adj_ < 0.05) compared to the values observed in age‐matched Normal control groups (Figure S6C–H).

### Dynamics of the microbial co‐occurrence network and functional metabolic pathways

**Figure 2:**
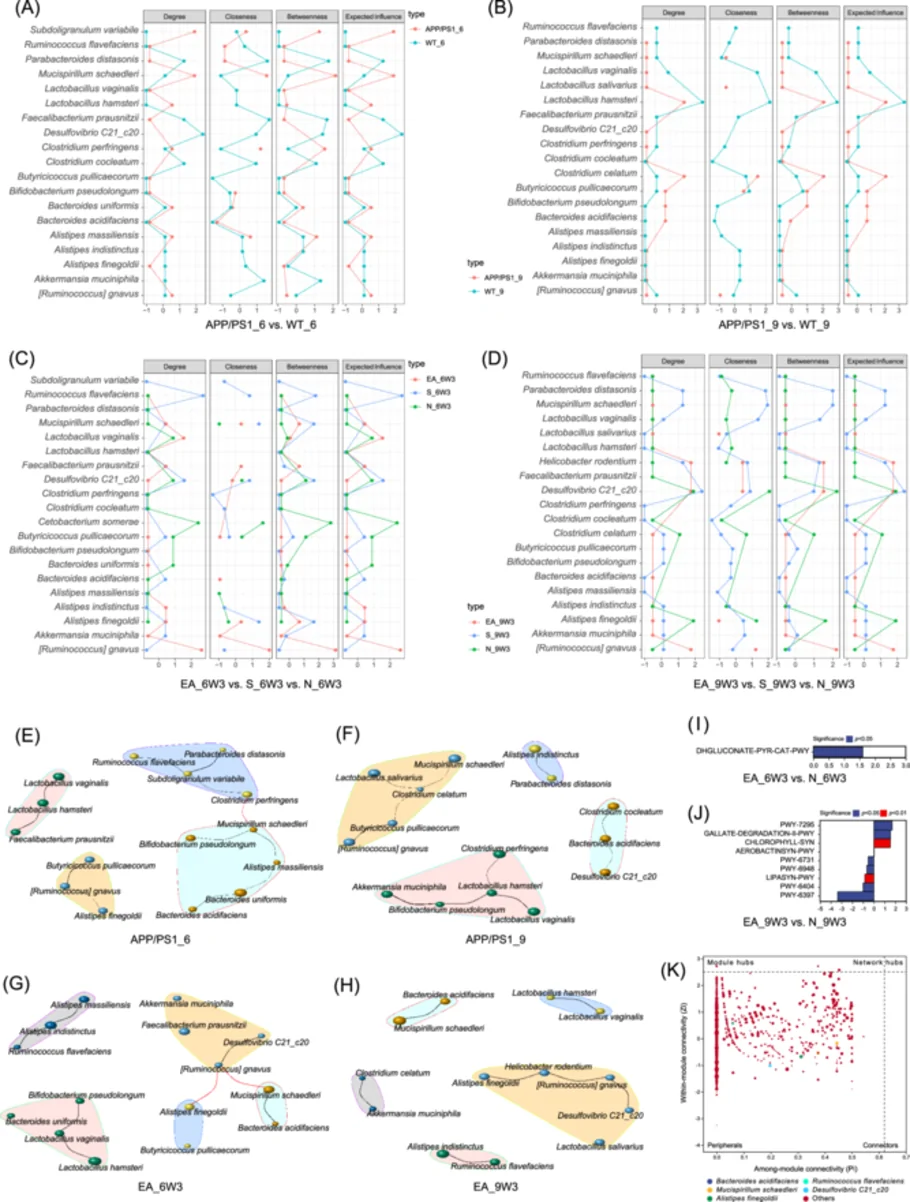
Network topography of core species and metabolic pathway variation pre‐ and post‐intervention. (A–D) Centrality indices, shown as standardized *Z*‐scores, indicate the interconnectivity between bacterial species. N, S, and EA represent the Normal, Sham, and Electroacupuncture groups, respectively, while 6W3 and 9W3 indicate the timepoints (i.e., 3 weeks after intervention in 6‐ and 9‐month‐old mice, respectively). (E–H) Co‐occurrence network of the 20 species with the highest relative abundance, as derived from Pearson's correlation coefficients using *Z*‐score standardization of log_2_‐transformed values. Nodes represent microbial species, and modules are annotated based on the proportion of their grouped composition. (I, J) Metabolic pathways exhibiting significant alterations following electroacupuncture. The *x*‐axis represents upregulation and downregulation based on the log_2_FC values. The *y*‐axis represents different pathways/group labels. (K) ZiPi plot depicting the distribution of nodes belonging to the top five microbial species with the highest abundance. The *x*‐ and *y*‐axes represent the Pi (among‐module connectivity) and Zi (within‐module connectivity) values, respectively. The thresholds are indicated by the dashed horizontal (Pi = 2.5) and vertical (Zi = 0.62) lines, which divide the microbes into four categories: network hubs, module hubs, connectors, and peripherals.

Keystone species were identified based on centrality scores and intra‐ and inter‐modular interactions within co‐occurrence networks, as generated by Pearson correlation coefficients (Figure 2A–H). Node centrality measures, including degree, closeness, betweenness, and expected influence, were utilized to highlight the modular structural features of each node. In the microbial co‐occurrence network of 6‐month‐old APP/PS1 mice, *Mucispirillum schaedleri* and *C. perfringens* were negatively correlated (*r* = −0.48, *p*_adj_ < 0.05; Figure S7A) and identified as keystone species, together with *Subdoligranulum variabile*, a diabetes‐related species (Figure 2A,E) [2]. Interestingly, *M. schaedleri* and *C. perfringens* also exhibited a significant negative correlation in the microbial co‐occurrence network of 6‐month‐old WT mice (*r* = −0.64, *p*_adj_ < 0.05; Figure S7B), while most other correlated pairs of microbes differed between 6‐month‐old APP/PS1 mice and WT mice.

Furthermore, in the microbial co‐occurrence network of 9‐month‐old APP/PS1 mice, *B. pseudolongum*, *L. hamsteri*, and *Butyricicoccus pullicaecorum*, which have anti‐inflammatory effects and produce short‐chain fatty acids, and *Clostridium celatum*, a risk factor for type 2 diabetes [3], were identified as keystone species (Figure 2B,F). Among them, *B. pseudolongum* and *L. hamsteri* exhibited a significant positive correlation (*r* = 0.66, *p*_adj_ < 0.05; Figure S7C), whereas *B. pullicaecorum* and *C. celatum* exhibited a significant negative correlation (*r* = −0.46, *p*_adj_ < 0.05; Figure S7C). In contrast, in the co‐occurrence network of 6‐ and 9‐month‐old APP/PS1 mice post‐electroacupuncture, *R. gnavus*, which is related to inflammatory bowel disease (IBD) and produces polysaccharides [4], was identified as a likely keystone species (Figure 2C,D,G,H). Notably, there was a larger proportion of significant positive correlations than negative correlations in the microbial co‐occurrence networks of 9‐month‐old APP/PS1 and WT mice (Figure S7C,D). PICRUSt2 analysis revealed that electroacupuncture significantly upregulated the DHGLUCONATE‐PYR‐CAT‐PWY: glucose degradation pathway in 6‐month‐old APP/PS1 mice (Figure 2I), as well as the PWY‐7295: L‐arabinose degradation IV, GALLATE‐DEGRADATION‐II‐PWY: gallate degradation I, and CHLOROPHYLL‐SYN: chlorophyllide a biosynthesis I pathways in 9‐month‐old APP/PS1 mice (Figure 2J).

### Discussion

Challenges to the conceptual “pathobiont” framework have emerged recently. Researchers have reported that an excess of beneficial bacteria may promote dysbiosis in the gut microbiome, and that certain microbial pathogens, despite their minor abundance, may also trigger inflammatory responses by remodeling benign microbiota. For example, *B. pseudolongum* has been shown to interact with diet‐derived cholesterol [5], and possesses significant potential for regulating lipid metabolism in obese mice and patients [6]. Two strains of *B. pseudolongum* (Bp7 and Bp8) have been demonstrated to modulate the intestinal epithelial barrier and alleviate colitis via the peroxisome proliferator‐activated receptor gamma/signal transducer and activator of transcription 3 (PPARγ/STAT3) pathway [7]. A recent study reported a significant increase in the abundance of *B. pseudolongum* and *L. vaginalis* in corticosterone‐treated mice exhibiting depression‐like behavior and reduced hippocampal neurogenesis [8].

From the perspective of animal ethology and translational medicine, each OFT indicator represents a certain phenotype of neuropsychiatric symptoms, which is influenced by the genotype of the rodent and the outcomes of the experimental model. However, BPSD‐associated disorders are not caused by a single bacterial species; rather, these disorders arise from complex interplay in the gut microbiota. The over‐abundance of *B. pseudolongum* in APP/PS1 mice may suggest the occurrence of “dysbacteriosis” characterized by significant deviations in the proportion of certain normal beneficial bacteria in the intestinal microbial ecosystem. This enhanced immune response to the naturally occurring gut microbiota of mice is strain‐specific, and variations in the abundance of *B. pseudolongum* may induce changes throughout the microbiome.

There is strong evidence for an inflammatory component in the pathogenesis of AD [9]. Despite contradictory findings relating to the pro‐ or anti‐inflammatory properties of *M. schaedleri* in IBD, such as Crohn's disease and colitis, a recent study suggested that the genus *Mucispirillum* may contribute to the underlying mechanisms of electroacupuncture in regulating the microbial composition of AD [10]. *C. perfringens* is an opportunistic pathogen that causes histotoxic and enterotoxic diseases [11]. *C. perfringens* type D epsilon toxin (ETX) causes severe neurological diseases that could lead to brain injury in ruminant livestock [12]. However, another study reported that the C‐terminal fragment of *C. perfringens* enterotoxin mutants 194 and m19 may play a key role in the treatment of AD by regulating insulin permeability via the mitogen‐activated protein kinases pathway [13]. Interestingly, *B. pseudolongum*, *M. schaedleri*, and *C. perfringens* formed a distinct cluster in the heatmap of 6‐ and 9‐month‐old EA‐treated group, highlighting the relationships among the key predictive microbes following electroacupuncture (Figure S3A,B). Given the importance of *M. schaedleri* for within‐module connectivity in the Zipi plot (Figure 2K), there is a clear need to further investigate the roles and interplay of *C. perfringens* and *M. schaedleri* in the co‐occurrence network of AD‐related BPSD.

Recent studies have shown that abnormal glucose metabolism may lead to mitochondrial dysfunction and oxidative stress in patients with AD and that improving glucose metabolism in the hippocampus may restore spatial memory in AD [14]. Increasing evidence supports the role of l‐arabinose and gallate in alleviating metabolic syndromes, such as obesity and hyperglycemia, as well as neurological disorders [15, 16]. l‐arabinose has also been shown to alleviate inflammatory damage to the intestines caused by infection with *Escherichia coli* O157:H7 [17]. Furthermore, chlorophyll has been demonstrated to mitigate neuronal cell death induced by PM 2.5 [18], while its derivative, chlorophyllide A, is used as a potent photosensitizer in photodynamic therapy, thus offering a promising alternative for the treatment of AD [19, 20]. Collectively, our findings support our hypothesis on the co‐regulation of glucose, l‐arabinose, and gallate degradation with chlorophyllide A biosynthesis in the gut microbiota of AD‐related BPSD following electroacupuncture intervention.

## 2 CONCLUSION

In conclusion, our findings provide novel insights into the profile of disease‐discriminative microbiota, the characteristics of electroacupuncture‐responsive bacteria, and their crosstalk with the host in microbial communities. Our findings suggest that electroacupuncture can ameliorate AD‐related BPSD by driving keystone species in the microbial co‐occurrence network and by regulating the composition and functional metabolic pathways of core microbiota.

## 3 METHODS

Please see the supporting information file for the methods section.

## AUTHOR CONTRIBUTIONS

**Fu‐You Su**: Conceptualization; methodology; data curation; formal analysis; validation; visualization; writing—review and editing; writing—original draft; project administration. **Chia‐Min Lin**: Formal analysis; visualization; validation. **Chao Liu**: Investigation. **Yiqin Yao**: Investigation. **Hui Wang**: Investigation. **Chunxue Zhang**: Investigation. **Wei Yi**: Supervision. **Nenggui Xu**: Supervision.

## CONFLICT OF INTEREST STATEMENT

The authors declare no conflicts of interest.

## ETHICS STATEMENT

The animal study was approved by the Animal Ethics Committee of Nanjing First Hospital, Nanjing Medical University (approval number: DWSY‐21072116). The study was conducted in accordance with the guidelines of the local legislation and institutional requirements.

## Supporting information


**Figure S1:** Distinct gut microbial signatures at baseline.
**Figure S2:** Baseline gut microbiota as a predictive biomarker.
**Figure S3:** Changes in gut microbiota structure and abundance following electroacupuncture.
**Figure S4:** Changes in the relative abundance distribution of gut microbiota following electroacupuncture.
**Figure S5:** Baseline differences of phenotypic and behavioral indicators.
**Figure S6:** Amelioration of BPSD‐like phenotype following electroacupuncture.
**Figure S7:** Relationship of microbial species using Pearson Correlation Coefficient.

**Table S1:** Microbial markers of 6‐month‐old APPswe/PS1δE9 (APP/PS1) mice identified by Linear discriminant analysis Effect Size.
**Table S2:** Microbial markers of 9‐month‐old APP/PS1 mice identified by Linear discriminant analysis Effect Size.
**Table S3:** Dysbiosis of gut microbiota in 6‐month‐old APP/PS1 mice.
**Table S4:** Dysbiosis of gut microbiota in 9‐month‐old APP/PS1 mice.
**Table S5:** Statistic methods.

## Data Availability

The data that support the findings of this study are openly available in NCBI at https://www.ncbi.nlm.nih.gov/bioproject/PRJNA1201107, reference number PRJNA1201107. Raw 16S rRNA sequencing data for samples used in this study have been deposited in the BioProject database at the National Center for Biotechnology Information (NCBI) under BioProject accession number PRJNA 1201107 (https://www.ncbi.nlm.nih.gov/bioproject/PRJNA1201107). The data used are saved in GitHub https://github.com/sufuyou2021/IMO-2025-0041. Supplementary materials (methods, figures, tables, graphical abstract, slides, videos, Chinese translated version, and update materials) may be found in the online DOI or iMeta Science http://www.imeta.science/imetaomics/.
