# Supplementary material for: Electroacupuncture reshapes the microbial co‐occurrence networks related to the behavioral and psychological symptoms of dementia in Alzheimer's disease
Source: IMetaOmics. 2025 Jul 2;2(4):e70035. doi: 10.1002/imo2.70035 (PMC12806058; doi:10.1002/imo2.70035)
Supplement: Supplementary file 1 — Figure S1: Distinct gut microbial signatures at baseline. Figure S2: Baseline gut microbiota as a predictive biomarker. Figure S3: Changes in gut microbiota structure and abundance following electroacupuncture. Figure S4: Changes in the relative abundance distribution of gut microbiota following electroacupuncture. Figure S5: Baseline differences of phenotypic and behavioral indicators. Figure S6: Amelioration of BPSD‐like phenotype following electroacupuncture. Figure S7: Relationship of microbial species using Pearson Correlation Coefficient. [file IMO2-2-e70035-s001.docx]

**Supporting Information to**

**Electroacupuncture reshapes the microbial co-occurrence networks related to** **the behavioral and psychological symptoms of dementia in Alzheimer’s disease**

**Running title: Acupuncture remodels gut microbiota in behavioral and psychological symptoms of dementia**

Fu-You Su^1^, Chia-Min Lin^2^, Chao Liu^3^, Yiqin Yao^3^, Hui Wang^3^, Chunxue Zhang^3^, Wei Yi^4^, Nenggui Xu^1^

^1^South China Research Center for Acupuncture and Moxibustion, Medical College of Acupuncture-Moxibustion and Rehabilitation, Guangzhou University of Chinese Medicine, Guangzhou, 510006, China

^2^Department of Electrical and Computer Engineering, Tamkang University, Taiwan, 251301, China

^3^Nanjing First Hospital, Nanjing Medical University, Nanjing, 210006, China

^4^The First Affiliated Hospital of Guangzhou University of Chinese Medicine, Guangzhou 510405, China

**Correspondence:** [fuyou_su@yeah.net; sufuyou@bpsd-research.org **(**Fu-You Su)](mailto:fuyou_su@yeah.net)

**METHODS**

**Animals**

A total of 72 male mice, including 48 APPswe/PS1ΔE9 mice, and 24 age-matched littermate C57BL/6J mice, were randomly divided into the EA (electroacupuncture), Sham, and Normal control groups (*n* = 12 in each age group), according to their body weights. All the mice were procured from Beijing Huafukang Biological Technology Company (Beijing, China) and acclimated for 10 days prior to experimentation. The mice were housed and maintained at 20–25°C on a 12:12 h day/night cycle, with food and water provided ad libitum. The experiment was conducted at Nanjing First Hospital, Nanjing Medical University. The animal ethics approval number was DWSY-21072116.

**Electroacupuncture Interventions**

The classic acupuncture point matching — Baihui (GV20) and bilateral Shenshu (BL23), derived from the “kidney-reinforcing and Du channel-regulating method,” was selected for electroacupuncture intervention in this study. The dilatational wave mode was selected for electroacupuncture, and the frequency and intensity were adjusted to 2 Hz/100 Hz and 1 mA, respectively. The acupuncture needle (0.25 × 13 mm, Suzhou Hwatuo Medical Instruments, China) was connected to the negative pole of a HANS-JS502A device and inserted unilaterally to a depth of approximately 2 mm at the GV20 region. Another needle was connected to the positive pole of the device and inserted slightly obliquely into the BL23 area to a depth of approximately 5 mm. The duration of treatment was set to 30 minutes, starting from when the power was turned on. Treatment was alternated every 15 minutes between the bilateral Shenshu (BL23) areas, while the other needle was left in the GV20 area during the entire treatment session. Considering the differential sensitivity to electrical stimulation in mice, the device control software was configured before the experiment to initiate the current at 0.1 mA and gradually increase it to 1 mA, thereby minimizing interference factors such as contextual fear conditioning. The intervention was administered once daily, with a one-day break after every 6 days, and continued for three consecutive weeks.

**Open Field Test**

The open field test (OFT) experiments were performed both pre- and post-intervention (*n* = 12 in each group, pre-intervention; and *n* = 8 in each group, post-intervention) for assessing the frequency of crossing the central zone, duration of time spent in the center, distance traversed in the central zone, and the total distance traveled. The illumination was set at 150 Lux, and the test was conducted from 13:00 to 16:00 (Beijing time). The mice were placed at the center of the OFT device and allowed to move freely for 5 minutes. Following the completion of testing for each mouse, the entire open-field arena was thoroughly cleaned to avoid leaving an abnormal odor. An OFT device (ZS-KC, 500 × 500 × 350 mm) and the Labmaze software, version 3.0 (Zhongshi Science and Technology Co., Ltd., Beijing, China), were used for the OFT experiments.

**Sample Collection**

Fecal samples were collected from each group at baseline and three weeks post-intervention. The samples collected over three consecutive days were pooled and immediately frozen in liquid nitrogen prior to storage at -80℃. The samples were then delivered on dry ice to the laboratory for microbial detection. The V3–V4 regions of the 16S rRNA genes were amplified using the 338F (5ʹ-ACTCCTACGGGAGGCAGCA-3ʹ) and 806R (5ʹ-GGACTACHVGGGTWTCTAAT-3ʹ) primers. The amplicon libraries were sequenced using an Illumina NovaSeq 6000 PE250 platform, with paired-end reads, at Personalbio Laboratory (Shanghai, China; <https://www.genescloud.cn>). The DADA2 method (Divisive Amplicon Denoising Algorithm 2) [[1](#_ENREF_1)] was used for filtering, denoising, removal of primer sequences, splicing and removal of chimerism.

**Statistical Analyses**

Statistical analyses were performed using R (versions 4.1.4 and 4.4.2), SPSS (version 25), and Python (version 3.12), and the QIIME2-based GENESCLOUD online platform (Table S5, [www.genescloud.cn](http://www.genescloud.cn/)) [[2](#_ENREF_2), [3](#_ENREF_3)]. The data from two independent groups were compared using the Mann-Whitney test, while data from three or more independent groups were compared using the Kruskal-Wallis test. The Wilcoxon signed-rank test was employed to compare the data obtained before and after EA interventions, followed by the Benjamini-Hochberg (BH) correction [[4](#_ENREF_4)]. The level of stringency was enhanced by minimizing the appearance of spurious correlations. To this end, the raw data were initially standardized in the following steps: (1) handling of zero values by adding a small constant, which represents a pseudo value (1.00E-20) commonly employed in microbial research, (2) standardization by transforming the values to log_2_ base, and (3) applying Z score standardization to the transformed values. Once the obtained variables satisfied the assumptions of normality, the Pearson correlation test was applied for correlation analysis [[5](#_ENREF_5)]. Statistical significance was determined using the following criterion: FDR < 0.05 following BH correction (^*^*p*_adj_ (adjusted *p* value) < 0.05, ^**^*p*_adj_ < 0.01, and ^***^*p*_adj_ < 0.001). The descriptive statistics were presented as the median [Q1–Q3], and the 20 most abundant bacterial species were reported in this study.

The microbes that exhibited significant variations, pre-and post-intervention with electroacupuncture, were determined using the Linear discriminant analysis Effect Size (LEfSe) method [[6](#_ENREF_6)] combined with analyses of the cladograms [[6](#_ENREF_6)], heatmaps [[7](#_ENREF_7)], boxplots [[8](#_ENREF_8)] , and volcano plots [[9](#_ENREF_9)]. The boxplots depicting the minimum, first quartile, median, third quartile and maximum abundance of the bacterial species were generated, using ggpubr and ggplot2 packages of R. The algorithm is based on standardization by transforming the values using the formula: log_2_(X+1) × 100. The linear discriminant analysis (LDA) plots and cladograms, which illustrated the hierarchical distribution of potential microbial markers, were generated using the built-in LEfSe Python and ggtree packages of R in the GENESCLOUD online platform, employing the one-against-all approach. The thresholds for LDA were set at 2, pre- and post-intervention. The distribution of microbial abundance was depicted using volcano plots, which were screened based on the following criteria: BH-FDR-adjusted *p* value < 0.05 and log_2_ Fold Change (FC) > 1.5 or < 1.5.

The clustering patterns of the major microbial species were depicted via heatmaps generated using the built-in pheatmap package of R in the GENESCLOUD online platform, based on Pearson correlation coefficients and Euclidean distance matrices. The composition of the core microbial communities at the species level was visualized with a stacked bar chart [[10](#_ENREF_10)], generated using the ggplot2 [[11](#_ENREF_11)] and cowplot packages of R. The similarities between the microbial communities were verified using orthogonal partial least squares-discriminant analysis (OPLS-DA) plots [[12](#_ENREF_12)], based on Euclidian distance matrices, generated using the built-in muma package of R in the GENESCLOUD online platform. The species-species associations inferred from Pearson correlation plots were generated using Python libraries, including pandas, numpy, seaborn , matplotlib , and scipy.stats [[13](#_ENREF_13)]. Random Forest models [[14](#_ENREF_14)] were additionally established to identify the important predictors of BPSD in Alzheimer’s Disease, using the built-in “classify_samples_ncv” function of the q2-sample-classifier (https://github.com/qiime2/q2-sample-classifier), based on the nested stratified cross-validation and 10-fold cross-validation methods in the GENESCLOUD online platform. Boxplot visualization of differences in behavioral indicators of the OFT [[15](#_ENREF_15)] was compared and visualized using Python libraries, including pandas, seaborn, and matplotlib.

Furthermore, microbial co-occurrence networks [[16](#_ENREF_16)] and centrality plots [[17](#_ENREF_17)] were generated using the corrplot, igraph, and qgraph packages of R for identifying the keystone species [[18](#_ENREF_18)] in the microbiomes of the different groups. The correlation coefficients cutoff R values to generate co-occurrence networks were 0.41 with *p*_adj_ < 0.05 for APP/PS1 and WT mice at baseline, and 0.57 with *p*_adj_ < 0.05 for all groups post-intervention. The module hubs, network hubs, connectors, and peripherals measured in log_2_ (CPM/n), were identified from ZiPi plots generated using the GENESCLOUD online platform. The alterations in the metabolic pathway modules of the core microbiota in APP/PS1 mice were predicted by PICRUSt2 (Phylogenetic Investigation of Communities by Reconstruction of Unobserved States) analysis [[19](#_ENREF_19)] , which aligns the sequences of 16S rRNA genes to the sequences in the MetaCyc [[20](#_ENREF_20)] and KEGG [[21](#_ENREF_21)] databases, using the built-in metagenomeSeq package of R in the GENESCLOUD online platform. The differences in the predictive metabolic pathway structures of the microbiota across the groups were additionally examined using Principal Coordinate Analysis (PCoA) plots [[22](#_ENREF_22)] with two dimensions, generated using the built-in vegan and ape packages of R in the GENESCLOUD online platform.

**REFERENCES**

1. Callahan, Benjamin J., Paul J. McMurdie, Michael J. Rosen, Andrew W. Han, Amy Jo A. Johnson, Susan P. Holmes. 2016. “DADA2: High-resolution sample inference from Illumina amplicon data.” *Nature Methods* 13: 581-583. <https://doi.org/10.1038/nmeth.3869>

2. Bolyen, Evan, Jai Ram Rideout, Matthew R. Dillon, Nicholas A. Bokulich, Christian C. Abnet, Gabriel A. Al-Ghalith, Harriet Alexander, et al. 2019. “Reproducible, interactive, scalable and extensible microbiome data science using QIIME 2.” *Nature Biotechnology* 37: 852-857. <https://doi.org/10.1038/s41587-019-0209-9>

3. Srivastava, Ankita, Yusuf Akhter, Digvijay Verma. 2024. “A step-by-step procedure for analysing the 16S rRNA-based microbiome diversity using QIIME 2 and comprehensive PICRUSt2 illustration for functional prediction.” *Archives of Microbiology* 206: 467. <https://doi.org/10.1007/s00203-024-04177-z>

4. Benjamini, Yoav, Yosef Hochberg. 1995. “Controlling the False Discovery Rate: A Practical and Powerful Approach to Multiple Testing.” *Journal of the Royal Statistical Society: Series B (Methodological)* 57: 289-300. <https://doi.org/https://doi.org/10.1111/j.2517-6161.1995.tb02031.x>

5. de Winter, Joost C., Samuel D. Gosling, Jeff Potter. 2016. “Comparing the Pearson and Spearman correlation coefficients across distributions and sample sizes: A tutorial using simulations and empirical data.” *Psychol Methods* 21: 273-290. <https://doi.org/10.1037/met0000079>

6. Khleborodova, Asya, Samuel D. Gamboa-Tuz, Marcel Ramos, Nicola Segata, Levi Waldron, Sehyun Oh. 2024. “lefser: implementation of metagenomic biomarker discovery tool, LEfSe, in R.” *Bioinformatics* 40: btae707. <https://doi.org/10.1093/bioinformatics/btae707>

7. Gu, Zuguang, Roland Eils, Matthias Schlesner. 2016. “Complex heatmaps reveal patterns and correlations in multidimensional genomic data.” *Bioinformatics* 32: 2847-2849. <https://doi.org/10.1093/bioinformatics/btw313>

8. Giorgi, Federico M., Carmine Ceraolo, Daniele Mercatelli. 2022. “The R Language: An Engine for Bioinformatics and Data Science.” *Life (Basel)* 12: 648. <https://doi.org/10.3390/life12050648>

9. Li, Wentian. 2012. “Volcano plots in analyzing differential expressions with mRNA microarrays.” *J Bioinform Comput Biol* 10: 1231003. <https://doi.org/10.1142/s0219720012310038>

10. Indratmo, Lee Howorko, Joyce Boedianto, Ben Daniel. 2018. “The efficacy of stacked bar charts in supporting single-attribute and overall-attribute comparisons.” *Visual Informatics* 2: <https://doi.org/10.1016/j.visinf.2018.09.002>

11. Gustavsson, Emil K., David Zhang, Regina H. Reynolds, Sonia Garcia-Ruiz, Mina Ryten. 2022. “ggtranscript: an R package for the visualization and interpretation of transcript isoforms using ggplot2.” *Bioinformatics* 38: 3844-3846. <https://doi.org/10.1093/bioinformatics/btac409>

12. Blasco, Helene, Jerzy Błaszczyński, Jean Charles Billaut, Lydie Nadal-Desbarats, Pierre François Pradat, David Devos, Caroline Moreau, et al. 2015. “Comparative analysis of targeted metabolomics: Dominance-based rough set approach versus orthogonal partial least square-discriminant analysis.” *Journal of Biomedical Informatics* 53: 291-299. [https://doi.org/10.1016/j.jbi.2014.12.001](https://doi.org/10.1016/j.jbi.2014.12.001)

13. McKinney, Wes. 2012. Python for data analysis: Data wrangling with Pandas, NumPy, and IPython. *" O'Reilly Media, Inc.* *"*, Sebastopol, CA, USA.

14. Cutler, Adele, D. Richard Cutler, John R. Stevens. 2012. Random Forests. *Ensemble Machine Learning: Methods and Applications* Springer New York, 157-175. <https://doi.org/10.1007/978-1-4419-9326-7_5>

15. Võikar, Vootele, S. Clare Stanford. 2023. The Open Field Test. *Psychiatric Vulnerability, Mood, and Anxiety Disorders: Tests and Models in Mice and Rats* Springer US, 9-29. <https://doi.org/10.1007/978-1-0716-2748-8_2>

16. Robinaugh, Donald J., Alexander J. Millner, Richard J. McNally. 2016. “Identifying highly influential nodes in the complicated grief network.” *Journal of Abnormal Psychology* 125: 747-757. <https://doi.org/10.1037/abn0000181>

17. Freeman, Linton C. 1978. “Centrality in social networks conceptual clarification.” *Social Networks* 1: 215-239. [https://doi.org/10.1016/0378-8733(78)90021-7](https://doi.org/10.1016/0378-8733(78)90021-7)

18. Berry, David, Stephanie Widder. 2014. “Deciphering microbial interactions and detecting keystone species with co-occurrence networks.” *Front Microbiol* 5: 219. <https://doi.org/10.3389/fmicb.2014.00219>

19. Douglas, Gavin M., Vincent J. Maffei, Jesse R. Zaneveld, Svetlana N. Yurgel, James R. Brown, Christopher M. Taylor, Curtis Huttenhower, Morgan G. I. Langille. 2020. “PICRUSt2 for prediction of metagenome functions.” *Nat Biotechnol* 38: 685-688. <https://doi.org/10.1038/s41587-020-0548-6>

20. Caspi, Ron, Richard Billington, Ingrid M. Keseler, Anamika Kothari, Markus Krummenacker, Peter E. Midford, Wai Kit Ong, Suzanne Paley, Pallavi Subhraveti, Peter D. Karp. 2020. “The MetaCyc database of metabolic pathways and enzymes - a 2019 update.” *Nucleic Acids Research* 48: D445-D453. <https://doi.org/10.1093/nar/gkz862>

21. Kanehisa, Minoru, Miho Furumichi, Mao Tanabe, Yoko Sato, Kanae Morishima. 2017. “KEGG: new perspectives on genomes, pathways, diseases and drugs.” *Nucleic Acids Research* 45: D353-d361. <https://doi.org/10.1093/nar/gkw1092>

22. Ayyala, Deepak Nag, Shili Lin. 2015. “GrammR: graphical representation and modeling of count data with application in metagenomics.” *Bioinformatics* 31: 1648-1654. <https://doi.org/10.1093/bioinformatics/btv032>

Figure S1. Gut microbial signatures at baseline. Cladograms showing variations in the abundance of gut microbiota between the groups of (A) 6- and (B) 9-month-old mice (Normal, Sham and Electroacupuncture (EA) groups). The circles radiating from the inside to outside represent the classification levels (from phylum to species).

Figure 2. Baseline gut microbiota as a predictive biomarker. Boxplots highlighting significant differences in gut microbiota between (A) 6- and (B) 9-month-old APP/PS1 and age-matched WT mice at baseline. (C, D) Random Forest plots depicting the top optimal microbial markers for discriminating between APP/PS1 and WT mice.

Figure S3. Changes in gut microbiota structure and abundance following electroacupuncture.

(A, B) Heatmaps depicting the significant differences in the clustering patterns of gut microbiota in mice aged 6 and 9 months, post-intervention with electroacupuncture. (C–F) Linear discriminant analysis plots and cladograms depicting the significant differences in the hierarchical relationships among the enriched taxa in the respective groups, post-intervention. (G) Stacked bar depicting the composition of microbiota in all the groups, pre- and post-intervention.

Figure S4. Changes in the relative abundance distribution of gut microbiota following electroacupuncture. (A–D) Volcano plots depicting the differential abundance distribution of gut microbiota between the EA-treated and control groups, and between the Sham and control groups aged 6 and 9 months. The bacteria that did not exhibit significant differences in abundance are denoted as gray dots. (E–H) Orthogonal partial least squares-discriminant analysis plots depicting the differences in the composition of gut microbiota among the three groups.

Figure S5. Baseline differences of phenotypic and behavioral indicators. (A–D and E–H) Comparison of the OFT indices of APP/PS1 mice, aged 6 and 9 months (n = 24 per group), with those of age-matched WT mice (n = 12 per group).

Figure S6. Amelioration of BPSD-like phenotypes following electroacupuncture. (A–D and E–H) Comparison of the OFT indices of the 6- and 9-month-old EA-treated groups post-intervention, with those of the age-matched Sham and control groups (n = 8 per group).

Figure S7. Relationship of microbial species using Pearson Correlation Coefficient. Bolded square borders represent significant correlations (*p*_adj_ < 0.05) in (A, B) 6- and (C, D) 9-month-old APP/PS1 mice and age-matched WT mice. Positive and negative correlations are indicated in pink and blue, respectively.
